# Collaborative Representation Using Non-Negative Samples for Image Classification

**DOI:** 10.3390/s19112609

**Published:** 2019-06-08

**Authors:** Jianhang Zhou, Bob Zhang

**Affiliations:** PAMI Research Group, Department of Computer and Information Science, Faculty of Science and Technology, University of Macau, Taipa, Macau 999078, China; mb85405@um.edu.mo

**Keywords:** collaborative representation-based classification, non-negative samples, image classification

## Abstract

Collaborative representation based classification (CRC) is an efficient classifier in image classification. By using $l_{2}$ regularization, the collaborative representation based classifier holds competitive performances compared with the sparse representation based classifier using less computational time. However, each of the elements calculated from the training samples are utilized for representation without selection, which can lead to poor performances in some classification tasks. To resolve this issue, in this paper, we propose a novel collaborative representation by directly using non-negative representations to represent a test sample collaboratively, termed Non-negative Collaborative Representation-based Classifier (NCRC). To collect all non-negative collaborative representations, we introduce a Rectified Linear Unit (ReLU) function to perform filtering on the coefficients obtained by $l_{2}$ minimization according to CRC’s objective function. Next, we represent the test sample by using a linear combination of these representations. Lastly, the nearest subspace classifier is used to perform classification on the test samples. The experiments performed on four different databases including face and palmprint showed the promising results of the proposed method. Accuracy comparisons with other state-of-art sparse representation-based classifiers demonstrated the effectiveness of NCRC at image classification. In addition, the proposed NCRC consumes less computational time, further illustrating the efficiency of NCRC.

## 1. Introduction

Image classification techniques have been extensively researched in computer vision [1,2,3,4,5]. Among them, sparse representation based classification methods [6] and its variants are frequently proposed and refined due to their effectiveness and efficiency, especially in face recognition [7,8,9]. Rather than using sparse representation (SR), the collaborative representation based classifier was proposed by using collaborative representation (CR), which achieved competitive performances with higher efficiency. The main difference between sparse representation and collaborative representation is the usage of different regularization terms in the minimization formulation, which is $l_{1}$ norm for sparse representation and $l_{2}$ norm for collaborative representation. Many applications have shown that both methods provide good results in image classification [5,10,11,12,13], where they can be further improved for a better recognition performance.

To improve the recognition rate, many works focus on weighting the training samples in different ways. For example, Xu et al. proposed a two-phase test sample sparse representation method [7] by using sparse representation in the first phase, followed by representation based on previously exploited neighbors of the test sample in the second phase. Timofte et al. imposed weights on the coefficients of collaborative representation [14] and achieved better performances in face recognition. Similarly, Fan et al. [8] provided another weights-imposing method, which derives weights of each coefficient from the corresponding training sample by calculating the Gaussian kernel distance between each training sample and test sample. In [7,8,14], the authors intended to seek a weight that can truly help in the classification, indicating that using different training samples may influence the discriminative pattern. Moreover, the negative coefficient implies a negative correlation between the training sample and test sample. Inspired by this idea, and considering the relationship between negative coefficients in the training sample and test samples, we intend to represent the test sample using non-negative representation. Ultimately, this produces a more effective classification [15,16,17].

The CRC uses $l_{2}$ norm to perform classification. As representation coefficients can be derived from an analytic solution to a least square problem, CRC is much more efficient than SRC. Furthermore, the collaborative representation is interpretive as well [18]. Due to its intrinsic property, CRC has been extensively refined. For instance, Dong et al. used sparse subspace on weighted CRC [14] to improve the recognition rate for face recognition [19]. Zeng et al. proposed S*CRC to achieve promising performances by fusing coefficients from sparse representation with the coefficients from collaborative representation [20]. However, in this work, each test sample is represented twice using SRC and CRC simultaneously, which is time consuming. Zheng et al. selected *k* candidate classes before representing a test sample collaboratively, while the *p* in the objective function should be defined in advance to obtain optimal results [21]. In [14,18,19,20,21], the authors ignored the relationship between the collaborative coefficient and the test sample. However, we strongly believe that, by considering this relationship, we are able to obtain a higher recognition rate.

The Restricted Linear Unit (ReLU) function [22] is widely used in deep learning to improve the performance of a deep neural network. Until now, many popular deep neural networks use ReLU as the activation function (e.g., VGG19 [3]). By using the ReLU function, there is sparse activation, making the network sparser. Similarly, we can apply the ReLU function to enhance the sparsity of CRC, which should improve its performance. Since the sparsity can help the CRC model to perform robust classification [23], representations mapped by the ReLU function will also achieve promising classification results.

**Figure 1:**
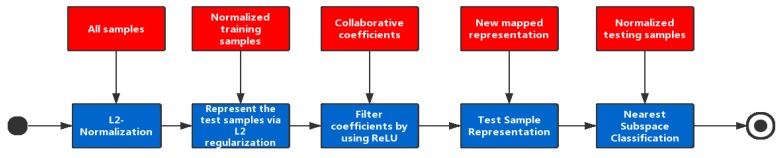
The pipeline of NCRC. In the first step, $l_{2}$ normalization is performed on all samples. Next, we calculate the collaborative representation coefficients by representing the test samples collaboratively via $l_{2}$ regularization using all training samples. In the third step, the ReLU function is utilized to filter collaborative coefficients and to map the negative ones to zero. Afterwards, we use the newly mapped collaborative coefficients to represent the test sample. In the last step, the nearest subspace classification is performed to classify each test sample.

Bringing everything together, this paper proposes a novel collaborative representation based classification method, named Non-negative CRC (NCRC). The system architecture of NCRC can be found in Figure 1. In the first step, NCRC performs normalization on all samples from a dataset based on $l_{2}$ norm. Next, NCRC uses all training samples to calculate the collaborative representation coefficients by representing the test samples collaboratively via $l_{2}$ regularization. Afterwards, the ReLU function is utilized to filter these collaborative coefficients and map the negative ones to zero. Then, we use the newly mapped collaborative coefficients to represent the test sample. Finally, to classify each test sample, the nearest subspace classification is performed. Specifically, the residuals between the representation of each class and the test sample are calculated, and we select the class label associated with the minimum residual as the result. The main contributes are:

1. We propose a novel image classification algorithm using non-negative samples based on the collaborative representation based classifier.
2. The proposed method enhances the sparsity of CRC by introducing the Restricted Linear Unit (ReLU) function, which increases the sparsity of the coefficients and improves the recognition rate.

The remainder of this paper is organized as follows. In Section 2, we give a brief overview of the collaborative representation based classifier before introducing our proposed NCRC. In Section 3, we show extensive experimental results to demonstrate the effectiveness and efficiency of NCRC, while at the same time discussing the superiority of our method. Finally, in Section 4, we give a conclusion.

## 2. CRC and Non-Negative CRC

### 2.1. Collaborative Representation-Based Classifier

Firstly, we review the background knowledge of the collaborative representation-based classifier. The collaborative representation based classifier [24] has been widely used in image classification, especially for face recognition [25,26,27]. Given *M* classes of samples and denote $X=\left[X_{1},X_{2},\ldots ,X_{N}\right]$ as the dataset, we calculate the collaborative coefficients as follows:

(1) $$\hat{\alpha }=\arg\min\alpha {∥y-X\alpha ∥}_{2}^{2}+\lambda {∥\alpha ∥}_{2}^{2}$$

where α is the collaborative coefficient vector, *y* is the test sample, and λ is the regularization term. According to Equation (1), we can obtain an analytic solution of $\hat{\alpha }$ as follows:

(2) $$\hat{\alpha }={\left(X^{T}X+\lambda I\right)}^{-1}X^{T}y$$

Lastly, the identity of test sample is determined by the minimum distance with a specific class subspace:

(3) $$identity\left(y\right)={\arg\min}_{i}{∥y-X_{i}\alpha_{i}∥}_{2}$$

We summarize CRC in Algorithm 1.

**Algorithm 1** Collaborative representation based classifier.
1:Normalize X to have $l_{2}$ norm
2:Calculate the collaborative representation coefficients vector $\hat{\alpha }$ using
$\hat{\alpha }=\arg\min\alpha {∥y-X\alpha ∥}_{2}^{2}+\lambda {∥\alpha ∥}_{2}^{2}$

3:Calculate the the residuals between test sample y and the representation of each class using formulation and obtain the identity of y using Equation (3):
$identity\left(y\right)={\arg\min}_{i}{∥y-X_{i}\alpha_{i}∥}_{2}$


### 2.2. Non-Negative Collaborative Representation Classifier

Based on CRC described in Section 2.1, we propose our methodology here. Since the collaborative representation based classifier uses positive as well as negative coefficients simultaneously to represent a test sample. The distribution of coefficients is not sparse enough, which makes the residuals of each class less discriminative. The negative coefficient indicates negative correlation between the test sample and its representation. Accordingly, we propose a novel collaborative representation, which only uses the non-negative representation, namely Non-negative Collaborative Representation based Classifier (NCRC). The proposed NCRC first represents the test sample using $l_{2}$ norm as follows:

(4) $$y=\alpha_{1}x_{1}+\alpha_{2}x_{2}+\alpha_{3}x_{3}+\ldots +\alpha_{n}x_{n}$$

where $\alpha =\left[\alpha_{1},\alpha_{2},\ldots ,\alpha_{n}\right]$ is obtained from Equation  (1)

Then, we introduce the Rectified Linear Unit (ReLU) function to filter the coefficient vector α:

(5) $$f\left(x\right)=\left\{\begin{array}{cc} x & ,x⩾0\\ 0 & ,x<0 \end{array}\right.$$

Afterwards, we represent the test sample using representation filtered by the ReLU function as follows:

(6) $$y=\tilde{\alpha_{1}}x_{1}+\tilde{\alpha_{2}}x_{2}+\tilde{\alpha_{3}}x_{3}+\ldots +\tilde{\alpha_{n}}x_{n}$$

Finally, the identity of a test sample is determined by calculating the residual between the test sample and each class:

(7) $$identity\left(y\right)={\arg\min}_{i}{∥y-X_{i}\tilde{\alpha_{i}}∥}_{2}$$

We summarize the NCRC classification procedure in Algorithm 2.

**Algorithm 2** Non-negative collaborative representation based classifier.
 1:Normalize X to have $l_{2}$ norm
 2:Calculate the collaborative representation coefficients vector $\hat{\alpha }$ using formulation (1)
$\hat{\alpha }=\arg\min\alpha {∥y-X\alpha ∥}_{2}^{2}+\lambda {∥\alpha ∥}_{2}^{2}$

 3:Use ReLU function described in Equation (5) to map each collaborative representations to non-negative representation
$f\left(x\right)=\left\{\begin{array}{cc} x & ,x⩾0\\ 0 & ,x<0 \end{array}\right.$

$\tilde{\alpha }=f\left(\hat{\alpha }\right)$

 4:Represent the test sample using non-negative representations described as Equation (6)
$y=\tilde{\alpha_{1}}x_{1}+\tilde{\alpha_{2}}x_{2}+\tilde{\alpha_{3}}x_{3}+\ldots +\tilde{\alpha_{n}}x_{n}$

 5:Calculate the the residuals between test sample y and representation of each class using Equation (7)
$identity\left(y\right)={\arg\min}_{i}{∥y-X_{i}\tilde{\alpha_{i}}∥}_{2}$


**Figure 2:**
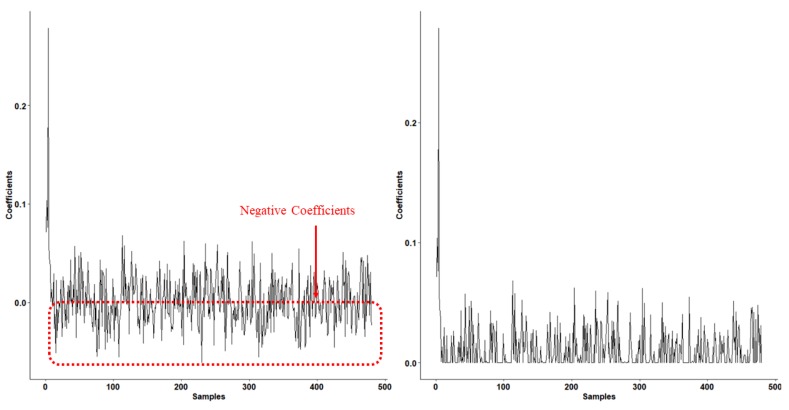
Coefficients of CRC (**left**) and NCRC (**right**) on an image from the AR database. Values on the horizontal axis represent the index of a sample in the training set. Values on the vertical axis represent the coefficient value. Samples (**left**) with negative (<0) coefficients are enclosed in a dotted red rectangle, indicating several coefficients still contain many negative values. However, these negative coefficients are non-existent on the right.

Figure 2 depicts the coefficients of each training sample (corresponding to the AR database; please refer to Section 3.1 for more information) from CRC (left) and NCRC (right), respectively. Since the test sample from the AR database belongs to the first class, it is obvious that training samples from the first class have a high positive value in this figure, indicating the high positive correlation with the test sample. It can also be observed from the CRC coefficient figure (left) that several coefficients still contain many negative values, which are samples having negative correlation with the test sample. Clearly, by using the ReLU filter, coefficients from NCRC (right) are much sparser than that of CRC. Obviously, several regions are sparser in NCRC compared with CRC (237 coefficients from NCRC are 0). As the sparsity of collaborative representation can help the classifier to perform more robust classification [20,23], the recognition performance of NCRC is better than the original CRC (see Section 3.2, Section 3.3, Section 3.4 and Section 3.5).

## 3. Experiments

We performed experiments on AR [28], LFW [29], MUCT [30] and the PolyU palmprint [31] datasets to verify the effectiveness of our method. To show our proposed method’s capability in different image classification tasks, the recognition tasks ranged from human face recognition to palmprint recognition, where the performances were validated by the hand-out method [32]. For each dataset, we divided them into training and testing to evaluate the result in each iteration. Besides this, we increased the size of the training samples in each iteration. At last, we computed the average accuracy achieved by the proposed method and compared it with other state-of-the-art sparse representation based classifiers (S*CRC [20] and ProCRC [18]), the original SRC [4] and CRC [24] as well as traditional classifiers such as SVM and KNN. To achieve the optimal results for all classifiers, various parameters were tested. For SRC, CRC, S*CRC, and ProCRC, we used λ = 1 × $e^{-3}$, 1 × $e^{-2}$, 1 × $e^{-1}$, 2 × $e^{-1}$, 3 × $e^{-1}$, and 4 × $e^{-1}$. In SVM, we tried different kernel functions, as for KNN (K = 7). All of the experiments were performed on a PC running Windows 10 with a 3.40 GHz CPU and 16 GB RAM running MATLAB R2018a. Below, for the comparison methods, we report the accuracy that was achieved using the optimal parameter. To guarantee the stability of our final results on each dataset, we repeated each experiment 30 times and took the average value as the final result.

### 3.1. Dataset Description

**Figure 3:**
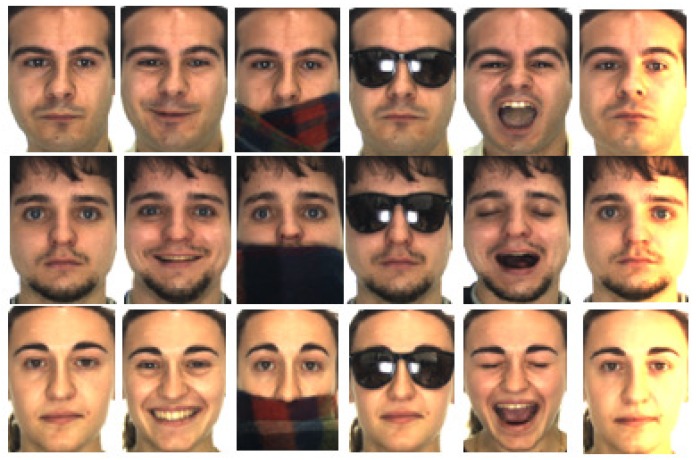
Image samples from the AR face database.

The AR face database [28] contains 4000 color images of 126 human faces, where each image is 768×576 pixels. Images of the same person are captured in two sessions separated by 14 days. In the experiments, we varied the number of training samples from 4 to 20 images per class, and took remaining samples in each class as the testing samples. Examples of images from this database can be found in Figure 3.

**Figure 4:**
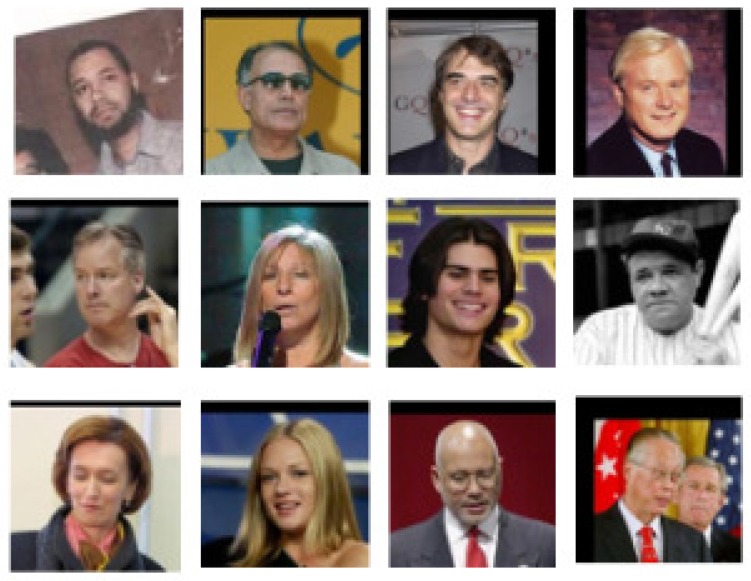
Image samples from the LFW face database.

The LFW face database [29] contains 13,233 images of 5749 human faces. Among them, 1680 people had more than two images. Figure 4 depicts some examples from this database. For the experiments, each image consisted of 32×32 pixels. We used the number of training samples from 5 to 35 images per class, and employed the remaining samples in each class as the testing samples. In addition, we applied FaceNet to extract the features from the database before feeding it into the classifiers.

**Figure 5:**
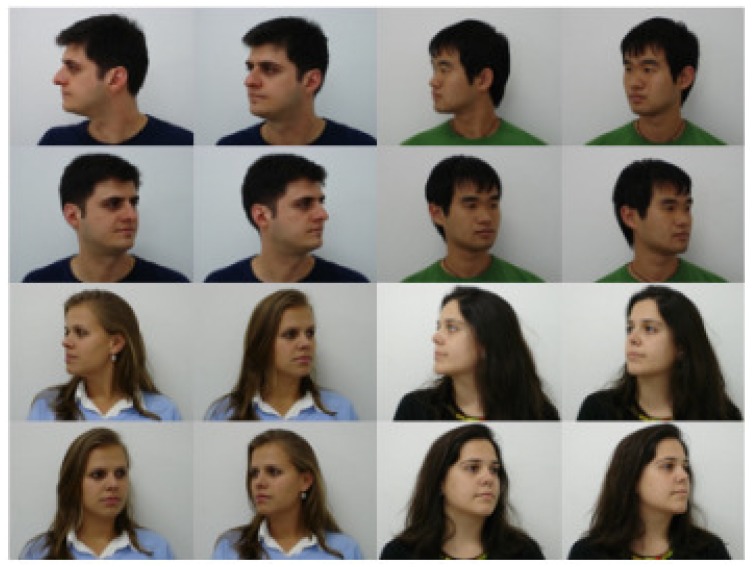
Image samples from the MUCT face database.

The MUCT face [30] database contains in total 3755 face images collected from 276 people. Each image is given a size of 640×480 pixels. These face images were captured by a CCD camera and stored in 24-bit RGB format. Samples from this database are shown in Figure 5. In the experiments, we adjusted the number of training samples from 1 to 7 in each class and took the remaining samples in each class as the testing samples.

**Figure 6:**
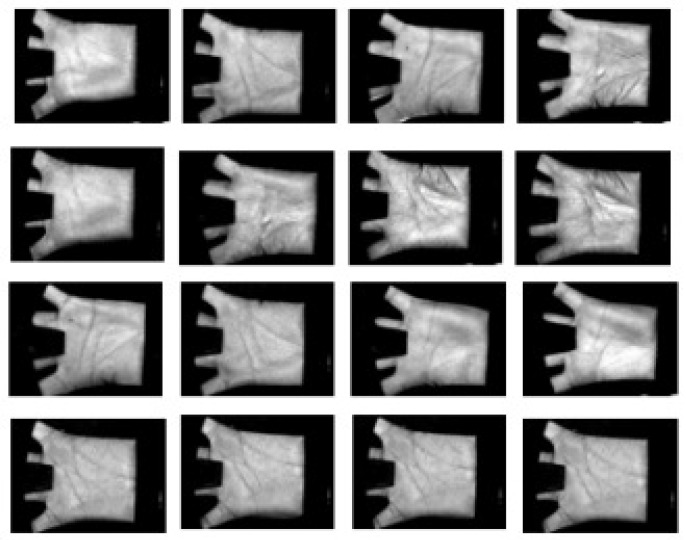
Image samples from the PolyU palmprint database.

The PolyU palmprint database was created by the Biometric Research Centre, Hong Kong Polytechnic University [31]. There are 7752 gray-scale images of 386 different palms stored in BMP format. Figure 6 illustrates some examples from this database. For an individual’s palmprint, there are approximately 20 samples collected in two sessions, where each palm image is 384×284 pixels. For our experiments, the number of training samples ranged from 1 to 5 images per class, and we used the remaining samples in each class as the testing samples.

The general information of the databases in the experiments is summarized in Table 1.

### 3.2. Experiments on the AR Face Database

**Figure 7:**
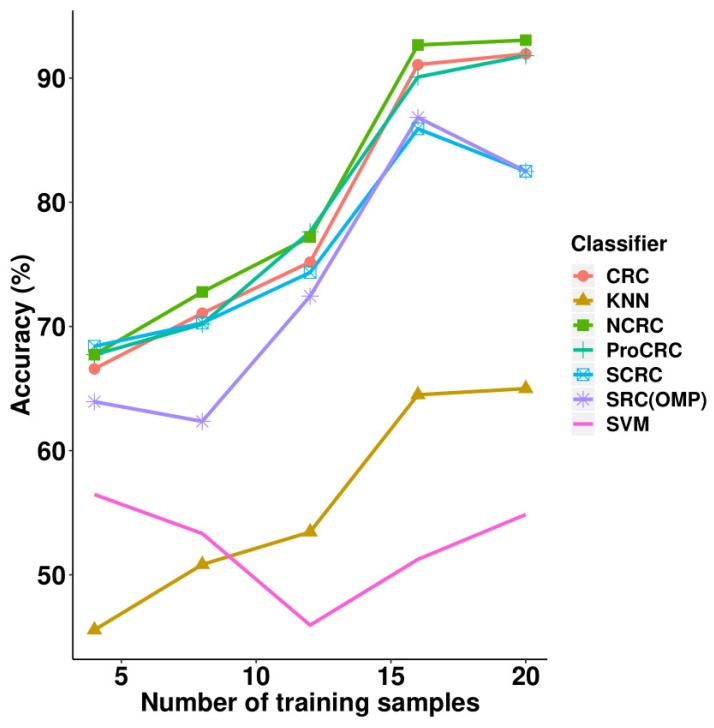
Accuracy vs. increasing the number of training samples on the AR database.

The experimental results on the AR face database can be found in Table 2 and Figure 7, where it can be seen that NCRC outperformed other classifiers. The highest recognition rate is 93.06% when using 20 samples per class for training. Compared with CRC, the improvement of NCRC ranges from 0.84% to 2.02%, which shows NCRC enhanced the recognition ability of the original CRC classifier on the AR database. Besides CRC, NCRC also achieved a better result than SRC (82.50%). Furthermore, the proposed method outperformed other variants of SRC and CRC such as S*CRC (82.50%, λ=0.01) and ProCRC (91.81%, λ=0.01), as well as traditional classifications including KNN (K = 7) and SVM (polynomial kernel function). For the parameter selection of NCRC, we experimented with λ = 1 × $e^{-3}$, 1 × $e^{-2}$, 1 × $e^{-1}$, 2 × $e^{-1}$, 3 × $e^{-1}$, and 4 × $e^{-1}$, respectively (refer to Table 3) by fixing the number of training samples at 20 (which was used to achieve the highest recognition). According to Table 3, NCRC obtained the best accuracy when λ=0.01. In Table 2, the highest accuracy achieved using each number of samples is marked in bold. In Table 3, highest accuracy achieved in each parameter λ is marked in bold.

### 3.3. Experiments on the LFW Face Database

**Figure 8:**
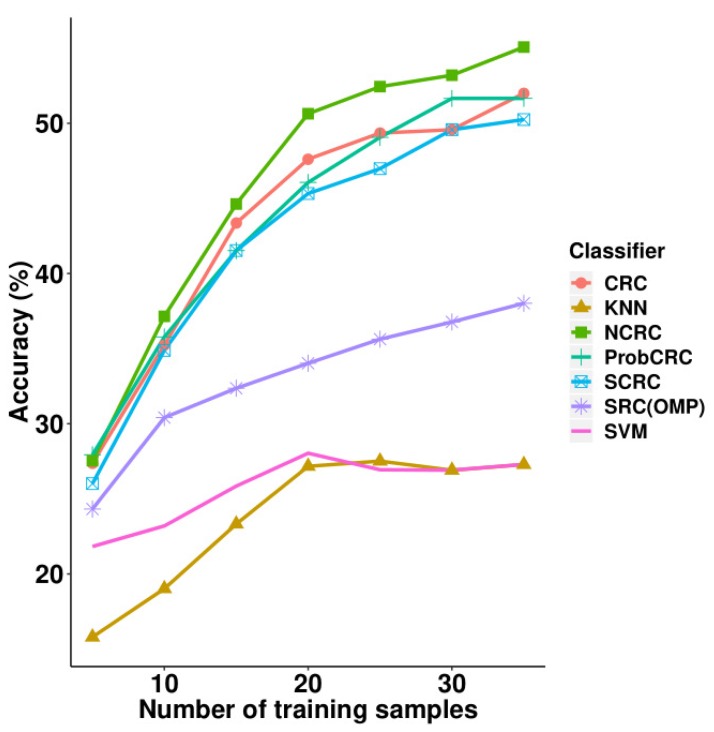
Accuracy vs. increasing the number of training samples on the LFW database.

Next, we performed experiments on the LFW face database and compared its results with other classifiers including SRC, CRC, S*CRC, ProCRC, SVM and KNN. These results are illustrated in Table 4 and Figure 8, where it can be observed that NCRC performed best using 10–35 training samples. When using five training samples, ProCRC slightly outperformed NCRC by 0.39%. For the parameter selection of NCRC, we experimented with λ = 1 × $e^{-3}$, 1 × $e^{-2}$, 1 × $e^{-1}$, 2 × $e^{-1}$, 3 × $e^{-1}$, and 4 × $e^{-1}$ (refer to Table 5) by fixing the number of training samples at 35. The optimal result was obtained when λ=0.1. Compared with CRC, the improvement of NCRC ranges from 0.17% to 3.62%, which shows NCRC again enhanced the recognition ability of the original CRC. The proposed method using 35 training samples also achieved a better result than SRC (38.02%). Furthermore, NCRC outperformed other variants of SRC and CRC such as S*CRC (50.25%, λ=0.1), ProCRC (51.66%, λ=0.01), SVM (28.12%, polynomial kernel function), and KNN (27.29%, K = 7). In Table 4, the highest accuracy achieved using each number of samples is marked in bold. In Table 5, highest accuracy achieved in each parameter λ is marked in bold.

### 3.4. Experiments on the MUCT Face Database

**Figure 9:**
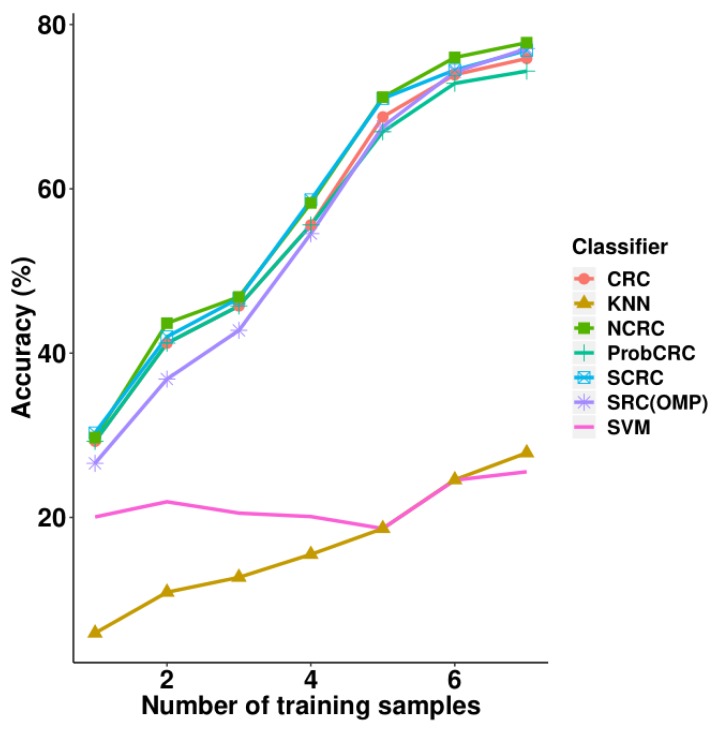
Accuracy vs. increasing the number of training samples on the MUCT database.

Table 6 and Figure 9 present the experimental results on the MUCT face database. From the seven different training sample sizes, NCRC attained the highest accuracy using 2–7 samples compared with the other classifiers. Using the same parameter selection progress as presented in Section 3.2 and Section 3.3 for NCRC, the best λ value using 7 training samples was 0.01 (refer to Table 7). The highest recognition rate is 77.78% when using seven samples per class for training. Compared with CRC, the improvement of NCRC ranges from 0.46% to 2.68%. Furthermore, NCRC also achieved a better result than SRC (77.07%), S*CRC (76.85%, λ=0.01) and ProCRC (74.32%, λ=0.01), as well as traditional classifications including KNN (K = 7) and SVM (polynomial kernel function). As for the use of one training sample, S*CRC outperformed NCRC by only 0.58%. In Table 6, the highest accuracy achieved using each number of samples is marked in bold. In Table 7, highest accuracy achieved in each parameter λ is marked in bold.

### 3.5. Experiments on the PolyU Palmprint Database

Finally, the experimental results on the PolyU palmprint database can be found in Table 8. According to this table, the highest average recognition rate with 1–5 training samples on average is 95.04% when using our proposed NCRC classifier, where various λ values were tested similar to the other experiments (refer to Table 9). Compared with CRC, the improvement of NCRC is 0.13% on average, which shows NCRC enhanced the recognition ability of the original CRC classifier on the PolyU palmprint database. When compared to the other classifiers, NCRC also achieved a better result than SRC (95.03%), Furthermore, the proposed method outperformed other variants of SRC and CRC such as S*CRC (94.92%), ProCRC (93.54%), KNN (57.99%), and SVM (86.91%). In Table 8, the highest average accuracy achieved is marked in bold. In Table 9, highest accuracy achieved in each parameter λ is marked in bold.

### 3.6. Comparison of Classification Time

**Figure 10:**
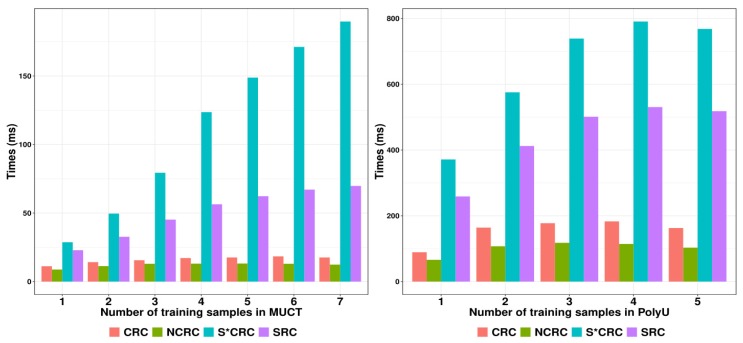
Comparison of the classification times using different databases with: MUCT (**left**); and PolyU (**right**).

To demonstrate the classification efficiency of the proposed NCRC, we further made comparisons between NCRC and other classifiers in terms of classification time. Figure 10 shows the classification time of SRC, CRC, S*CRC and NCRC on the MUCT (left) and PolyU (right) databases with an increasing number of training samples. As shown in Figure 10, NCRC required less classification time compared with the other classifiers, which indicates a higher efficiency for image classification. Even though the classification accuracy of MUCT using S*CRC with one training sample is slightly higher than the result of NCRC (refer to Table 6), in terms of classification time, NCRC performed (over three times) faster than S*CRC. As a variant of CRC, the ProCRC used less classification time than our proposed method, while its performance on the datasets show inferiority compared with our proposed method. These can be seen in Table 2, Table 4, Table 6 and Table 8, with NCRC outperforming ProCRC every time except on the LFW database using five training samples, where the difference is only 0.39%.

### 3.7. Discussion

We conducted experiments ranging from face to palmprint recognition, where it has been well proven that our proposed method achieved promising performances as well as efficient classification times. Here, we are able to reach the following inferences from the experiments:

1. For face recognition, NCRC tends to achieve better results when the number of training samples is increased compared with SRC and CRC. Here, the highest improvement reaches 17.3% on the LFW database. Furthermore, NCRC (AR: 93.06%; LFW: 55.32%; and MUCT: 77.78%) is even more effective in terms of accuracy than S*CRC (AR: 82.50%; LFW: 50.25%; and MUCT: 76.85%) and ProCRC (AR: 91.81%; LFW: 51.66%; and MUCT: 74.32%), which are refined classifiers based on SRC (AR: 82.50%; LFW: 38.02%; and MUCT: 77.07%) and CRC (AR: 91.94%; LFW: 52.00%; and MUCT: 75.86%).
2. When it comes to palmprint recognition, NCRC (95.04%) also shows competitive recognition rates on average, reaching its highest improvement of 1.5% over other state-of-the-art sparse representation methods such as SRC (95.03%), CRC (94.91%), S*CRC (94.92%), and ProCRC (93.54%). This indicates that our proposed method is not only effective in face recognition, but also other image classification tasks.
3. Besides the recognition rate, NCRC (MUCT: 12.1 ms) consumed less time in classification compared with SRC (MUCT: 50.9 ms), CRC (MUCT: 16.0 ms) and S*CRC (MUCT: 113 ms) implying its efficiency in image classification. Although ProCRC performed faster than NCRC, in terms of the recognition rate, the proposed method outperformed all classifiers on average.

## 4. Conclusions

In this paper, we propose a novel collaborative representation classifier termed as NCRC to perform image classification. When performing test sample representation, NCRC uses a ReLU function to enhance the sparsity of the CRC coefficients. Afterwards, the proposed method represents the test sample collaboratively using new non-negative coefficients. Finally, in the classification stage, the nearest subspace classifier is applied. According to extensive experiments, the proposed NCRC on average outperformed other popular classifiers in different image classification tasks ranging from face recognition to palmprint recognition using both recognition rate and classification time as performance measurements. This proves our new representation is effective as well as efficient. Therefore, the novel classifier has the potential to be applied to real recognition tasks that require a higher accuracy and faster recognition speeds. As part of our future work, we will extend this method using non-negative representation for classification to other classifiers to eventually develop a deep learning based methodology.

## Figures and Tables

**Table 1:** Information of the databases.

Database	Classes	Samples	Image Size	Dimension
AR	120	3120	40×32	2-D
LFW	86	1251	32×32	2-D
MUCT	276	3755	640×480	2-D
PolyU Palmprint	386	7752	384×284	2-D

**Table 2:** Recognition performance of NCRC and the comparison methods on the AR database.

Samples	SRC	CRC	S*CRC	ProCRC	SVM	KNN	NCRC
4	63.94	66.59	**68.40**	67.72	56.47	45.57	67.73
8	62.36	71.06	70.28	70.19	53.33	50.83	**72.78**
12	72.44	75.18	74.34	77.62	45.95	53.45	**77.20**
16	86.83	91.83	85.91	90.08	51.25	64.50	**92.67**
20	82.50	91.94	82.50	91.81	54.86	65.00	**93.06**

**Table 3:** Parameter selection of NCRC for the AR database.

λ	0.001	0.01	**0.1**	0.2	0.3	0.4
**Accuracy**	92.50	**93.06**	91.94	91.67	90.56	90.42

**Table 4:** Recognition performance of NCRC and the comparison methods on the LFW database.

Samples	SRC	CRC	S*CRC	ProCRC	SVM	KNN	NCRC
5	24.32	27.37	26.02	**27.93**	21.83	15.80	27.54
10	30.41	35.24	34.88	35.78	23.20	19.02	**37.15**
15	32.36	43.36	41.53	41.53	25.85	23.32	**44.62**
20	34.03	47.61	45.33	46.07	28.04	27.17	**50.64**
25	35.63	49.35	46.98	51.66	26.94	27.51	**52.44**
30	36.78	49.58	49.58	51.66	28.14	26.91	**53.20**
35	38.02	52.00	50.25	51.66	28.12	27.29	**55.32**

**Table 5:** Parameter selection of NCRC on the LFW database.

λ	0.001	0.01	**0.1**	0.2	0.3	0.4
**Accuracy**	42.1	55.07	**55.32**	51.33	48.25	46.67

**Table 6:** Recognition performance of NCRC and the comparison methods on the MUCT database.

Samples	SRC	CRC	S*CRC	ProCRC	SVM	KNN	NCRC
1	26.58	29.26	**30.30**	29.26	20.06	5.92	29.72
2	36.84	41.21	42.00	41.21	21.91	10.90	**43.65**
3	42.77	45.75	46.67	45.75	20.53	12.70	**46.84**
4	54.55	55.60	58.66	55.60	20.11	15.50	**58.28**
5	67.58	68.76	70.99	66.95	20.59	18.65	**71.16**
6	74.23	73.89	74.46	72.84	24.54	24.58	**75.99**
7	77.07	75.86	76.85	74.32	25.56	27.87	**77.78**

**Table 7:** Parameter selection of NCRC on the MUCT database.

λ	0.001	0.01	**0.1**	0.2	0.3	0.4
**Accuracy**	74.49	**77.78**	74.60	71.80	69.12	67.2

**Table 8:** Average accuracy of NCRC and the comparison methods on the PolyU palmprint database.

SRC	CRC	S*CRC	ProCRC	SVM	KNN	NCRC
95.03	94.91	94.92	93.54	86.91	57.99	**95.04**

**Table 9:** Parameter selection of NCRC on the PolyU palmprint database.

λ	0.001	0.01	**0.1**	0.2	0.3	0.4
**Accuracy**	94.9	**95.04**	93.4	92.28	91.26	97

## Abbreviations

The following abbreviations are used in this manuscript:

SRC	Sparse representation-based classifier
CRC	Collaborative representation-based classifier
ProCRC	Probabilistic collaborative representation-based classifier
NCRC	Non-negative collaborative representation-based classifier
KNN	K nearest neighbor classifier
SVM	Support vector machine
ReLU	Restricted Linear Unit
